# A cross sectional study of the prevalence, risk factors and population attributable fractions for limb and body lesions in lactating sows on commercial farms in England

**DOI:** 10.1186/1746-6148-5-30

**Published:** 2009-08-24

**Authors:** Amy L KilBride, Claire E Gillman, Laura E Green

**Affiliations:** 1 Department of Biological Sciences, University of Warwick, Coventry, CV4 7AL, UK

## Abstract

**Background:**

Lesions on sows' limbs and bodies are an abnormality that might impact on their welfare. The prevalence of and risks for limb and body lesions on lactating sows on commercial English pig farms were investigated using direct observation of the sows and their housing.

**Results:**

The prevalence of lesions on the limbs and body were 93% (260/279) and 20% (57/288) respectively. The prevalence of limb and body lesions was significantly lower in outdoor-housed sows compared with indoor-housed sows. Indoor-housed sows had an increased risk of wounds (OR 6.8), calluses (OR 8.8) and capped hock (OR 3.8) on their limbs when housed on fully slatted floors compared with solid concrete floors. In addition, there was an increased risk of bursitis (OR 2.7), capped hock (OR 2.3) and shoulder lesions (OR 4.8) in sows that were unwilling to rise to their feet. There was a decreased risk of shoulder lesions (OR 0.3) and lesions elsewhere on the body (OR 0.2) in sows with more than 20 cm between their tail and the back of the crate compared with sows with less than 10 cm.

**Conclusion:**

The sample of outdoor housed sows in this study had the lowest prevalence of limb and body lesions. In lactating sows housed indoors there was a general trend for an increased risk of limb and body lesions in sows housed on slatted floors compared with those housed on solid concrete floors with bedding. Sows that were less responsive to human presence and sows that had the least space to move within their crates had an additional increased risk of lesions.

## Background

Outdoors, sows farrow in a hut with free access to an outdoor paddock. In indoor production, sows usually farrow within a farrowing crate in an individual pen. The crate floor must, as far as possible, meet all the sow's needs for a comfortable surface for lying, sufficient space and a non slip surface for rising and standing, separation from excreta and be robust to her size and weight. To meet these requirements and to minimise labour, farrowing pen floors are often slatted and have little or no bedding.

In farrowing pens the floor type has been associated with the risk of developing limb and body lesions. In 383 lactating sows on an experimental unit the lowest prevalence of limb wounds occurred in sows housed on solid concrete floors and the prevalence increased as the proportion of the pen that was slatted increased [1]. In pregnant sow housing, slatted floors, compared with solid floors, and absence of bedding have been reported to be associated with increased prevalence of bursitis [2] and calluses [3]. Slatted floors were associated with an increased risk of body lesions in 555 lactating sows from 10 commercial herds in Denmark [4]. Further risks for body lesions include small farrowing crates [5], poor body condition [4,6-9] and wet skin [4,6,9].

In this paper, the prevalence of, population attributable fractions for, and risks associated with limb and body lesions in a sample of lactating sows housed indoors and outdoors are presented.

## Methods

### Sample size

The data presented in this paper were collected as part of a larger study investigating the impact of floor type on commercially reared pigs of all ages. The farm selection criteria were breeder-to-finisher units with more than 100 breeding sows. In the absence of published studies on the prevalence of limb and body lesions in lactating sows in Britain, estimates from rearing pigs, were used [10]. Assuming 95% of herds have sows with limb and body lesions and an approximate population of 1,870 (number of herds fitting the selection criteria in 2003 in Britain according to DEFRA, personal communication) with 95% confidence intervals and 5% precision, it was calculated that it was necessary to sample sows from a minimum of 75 farms. A figure of 100 farms was chosen for the study design.

Assuming an approximate study population of 60,000 lactating sows on the target population farms (average herd size 220 sows according to DEFRA, 2003 data, personal communication), 50% lesion prevalence, a 95% confidence interval and 5% precision, with a farm level intraclass coefficient of 0.1 [11], it was calculated that a sample of approximately 400 lactating sows was required to estimate the prevalence of lesions if four sows were sampled from each of 100 farms. To detect a 3 fold difference in risk between exposed and unexposed sows with 95% confidence and 80% power given a 10% prevalence of disease in the unexposed sows, with an estimated farm intracluster correlation coefficient of 0.1, a sample size of approximately 250 sows was required. Sample size calculations were carried out in Win Episcope 2.0.

### Data collected

A total of 549 breeder-finisher pig farms in England and Wales with more than 100 breeding sows were randomly selected from the Assured British Pig (ABP) database and invited to participate in the study. A total of 101 farmers agreed to take part in the study (18% compliance); 7 of these farms were used to pilot test the recording systems and to train observers. Data on lactating sows were collected from 89 farms. There was only one farm located in Wales so this farm was excluded from calculations of prevalence and population attributable fractions (n = 88). The Welsh farm, and a further 9 farms that were non-randomly selected for participation (5 from Scotland, recruited by Quality Meat Scotland and 4 from England recruited via their veterinarian), were included in the risk factor analysis giving a total of 99 farms.

### Sow observations

On each farm, four lactating sows, one each at 3 - 7, 8 - 14, 15 - 21 and 22 - 28 days post partum, were randomly selected using random number tables (counting from the first pen/hut on the left of the entrance). A comprehensive protocol was written defining every lesion and score. Scoring systems were tested and compared on the seven on-farm training days prior to data collection. Eight trained researchers (all with science or agricultural degrees and experience with pigs) recorded data on the sows. All observers examined both indoor and outdoor housed sows.

For the purpose of classification; lesions dorsal to the elbow joint (condyle of humerus) on the fore limbs and the stifle (lateral condyle of femur) on the hind limbs were classed as body lesions while lesions ventral to these joints were classified as limb lesions. The sow's body and all four limbs were examined and all injuries were classified and their location and severity recorded (Table 1).

**Table 1:** Case and score definitions for limb and body lesions in lactating sows

Lesion	Definition	Score
Limb lesions		
Wounds	Loss of the outer epidermis resulting in an open wound or a healing wound with a scab	0 = no lesion, 1 = <25%, 2 = 25-50%, 3 = >50% of the size of the nearest joint on the diseased limb.
Calluses	^1^Alopecia and hypercalcification of the skin	
Bursitis	^2^Fluid filled sac in the subcutaneous tissue	
Capped hock	^2^Bursa swelling on the point of the hock joint on the hind limb	
Body lesions		
Old scar	Healed with no blood or scabs evident	0 = no scaring 1 = small scar (<2 cm) 2 = moderate scar (2-3 cm) 3 = large scar (>3 cm)
New lesion	Fresh, open or healing wounds with scabs	0 = no lesion 1 = redness/soreness where the surface of the skin is not broken or a small area of broken skin/scab (<2 cm) 2 = moderate area of broken skin/scab (2-3 cm) 3 = large area of broken skin/scab (>3 cm)

^1^[3]

^2^[13]

Observations were made of a sow's willingness to rise in the crate in response to human presence (Table 2) [12]. The sow's body condition was scored using DEFRA guidelines (Condition Scoring of Pigs, PB 3480). The size of sows in relation to the crate in which they were housed was assessed by estimating the space between the sow and the crate while the sow was standing with her snout in the feed trough. Sows were encouraged to root in their trough either by tapping the trough or by activating their drinker. The distance between a sow's back and the top of the crate was classified as less than 5 cm, 5-10 cm or more than 10 cm at this observation. The distance between a sow's tail and the back of the crate was classified as less than 10 cm, 10-20 cm or more than 20 cm.

**Table 2:** Classification of sow's initial response to human presence

Score	Classification^1^
0	Bright alert and responsive - sow rises immediately
1	Bright but less responsive - may remain down, or dog sitting, before eventually rising
2	May be dull - only rises when strongly motivated
3^2^	Dull and unresponsive - will not rise

^1^[12]

^2^Combined with score 2 for analysis due to the small number of sows in this category

### Farrowing pen observations

Data were collected on the floor type, the condition of the floor and the use of bedding (Table 3). Indoor farrowing pen floors were divided into three areas; the pen outside the crate, the anterior part of the floor inside the crate, here after referred to as the sow lying area, and the sow dunging area.

**Table 3:** Definition of variables collected on the farrowing pens of indoor housed lactating sows

Variable	Definition
Pen construction	
Floor type	Solid, partly slatted or fully slatted
Floor material	Soil, concrete, metal or plastic
Bedding	
Bedding location	Bedding only in the anterior part of the crate (lying area or bedding in all areas of the crate
Bedding type	Straw, wood shavings or paper
Floor condition	
Cleanliness	Wet
- presence or absence	Dry slurry
	Wet slurry
	Spilled food
	Fresh dung
Damage	Sharp edges
- presence or absence	Broken/cracked
	Worn rough surface

### Data checking and data analysis

A sow was defined as diseased if a lesion of score one or more (Table 1) was present. The prevalence of each different type of lesion was calculated separately. The maximum score per pig for each type of lesion was used in analysis of prevalence and associated risk factors.

The crude prevalence of each injury was calculated among the sows from the ABP English farms as follows:



The outcome variable used in the majority of risk factor analyses for each separate lesion was; 0 = sows with no lesions, 1 = sows with a lesion score 1-3. The exceptions were calluses and capped hock where, because of the high prevalence of calluses and the very mild nature of capped hock score one [14], diseased sows were classified as those with a lesion score of two or three.

Generalised binomial linear regression models with mixed effects were used with sows (level 1) nested within farms (level 2) to account for the similarity of sows within farms. MLwiN version 2.01 was used for all multilevel analysis [15]. The week of lactation was included in the models throughout the initial screening for all outcomes and forced into the final models regardless of statistical significance.

Models were built to compare indoor with outdoor housed sows. Separate models were built for sows housed indoors to investigate floor construction, bedding use and floor condition. Partly slatted floors with varying amounts of bedding were compared to investigate the effect of slat material and type of bedding on sow injury. The identity of the observer was tested in each final model to investigate whether it altered the interpretation of the fixed effects.

To check for a linear association with the outcome, continuous variables were categorised and the categories were examined for patterns of increasing or decreasing coefficients. Where the associations were non-linear the variables remained categorised. Variables were taken forward for multivariable analysis when significant at p < 0.2 [11]. Where variables were highly correlated, the most biologically plausible and useful variable, based on biological knowledge and previous research, was selected for inclusion in the model. Both forward addition and backward elimination were used to identify the variables that had a significant association (p < 0.05) with the outcome in the final model [11]. Finally, all variables rejected at the screening stage were retested in the final model to check for residual confounding [16].

The equation for the logistic models took the form:



Where p_ij _= the probability of a lactating sow having a particular lesion, investigated with a logit link function, β_0 _= constant, βx is a vector of fixed effects varying at level 1 (ij) or level 2 (j), i is lactating sows, j is farms and v_j _and u_ij _are the level two and level one residual variances for the distributions for sow and farm respectively.

Associations between the ordinal lesion score of different types of lesion were investigated using Pearson correlation. The Mann-Whitney U test was used to compare parity between indoor and outdoor housed sows. The Chi-square test was used to investigate whether lesion were more likely to be bilateral than by chance. The population attributable fractions for limb and body lesions were calculated from the ABP farms in England using the formula:



Where AF_p _is the population attributable fraction for each floor type, RD is the risk of a lesion in the exposed group minus the risk in the reference category group, p(E+) is the proportion of sows on a floor type and p(D+) is the proportion of sows with the lesion on a floor type [11]. Fractions are converted to percentages and summed across the floor types to calculate the total reduction possible if all sows were housed on the reference floor type.

## Results

### Herd, pen and sow characteristics

The study was carried out between 23 September 2003 and 2 August 2004. Visits to indoor and outdoor herds were spread throughout the year, 4-14 farms were visited per month. In total 328 sows were examined from 99 farms; 50 from 17 outdoor farms and 278 from 82 indoor farms. Prevalence estimates were calculated from sows from 16 outdoor and 73 indoor ABP English farms where complete data were available. See Tables 4, 5, 6, 7, 8, 9, 10, 11, 12 for exact n values. The median herd size of the ABP English farms was 320 (IQR 220, 475) breeding sows (mean = 409).

**Table 4:** Number and percent of 279 lactating sows from 86 indoor and outdoor English farms with limb lesions by limb and score

	Wounds		Calluses		Bursitis		Capped hock	
	n	%	n	%	n	%	n	%
Limb								
Right fore	18	6.5	222	79.6	31	11.1		
Left fore	19	6.8	225	80.6	26	9.3		
Right hind	23	8.2	68	24.4	66	23.7	150	53.8
Left hind	24	8.6	68	24.4	57	20.4	149	53.4
Bilateral								
Fore limbs	9^1^	3.2	216^2^	77.4	20^3^	7.2		
Hind limbs	15^4^	5.4	59^5^	21.1	43^6^	15.4	139^7^	49.8
Score^8^								
Score 0	228	81.8	42	15.0	176	63.1	120	43.0
Score 1	35	12.5	48	17.2	44	15.8	90	32.3
Score 2	14	5.0	102	36.6	45	16.1	60	21.5
Score 3	2	0.7	87	31.2	14	5.0	9	3.2

^1 ^χ^2 ^= 58.0, df = 1, p < 0.01. ^2^χ^2 ^= 196.1, df = 1, p < 0.01. ^3^χ^2 ^= 127.7, df = 1, p < 0.01.

^4^χ^2 ^= 88.8, df = 1, p < 0.01. ^5^χ^2 ^= 196.1, df = 1, p < 0.01. ^6^χ^2 ^= 118.5, df = 1, p < 0.01.

^7^χ^2 ^= 211.3, df = 1, p < 0.01.

^8^0 = no lesion, 1 = <25%, 2 = 25-50%, 3 = >50% of the size of the closest limb joint

**Table 5:** Number and percent of 288 sows from 86 indoor and outdoor English farms with new body lesions and body scars by body location

		New lesion^1^		Scars^2^		Any lesion	
Body location		n	%	n	%	n	%
Shoulder	Left	22	7.6	17	5.9	37	12.8
	Right	21	7.3	17	5.6	37	12.8
	Bilateral	13^3^	4.5	6	2.1		
Hip	Left	3	1.0	5	1.7	8	2.8
	Right	2	0.7	5	1.7	7	2.4
	Bilateral	1	0.3	2	0.7		
Tail		22	7.6	10	3.5	32	11.1
Spine		12	4.2	13	4.5	25	8.7
Hip, tail and spine total		33	11.4	28	9.7	60	20.8

^1^Fresh, open or healing wounds with scabs

^2^Healed with no blood or scabs evident

^3^More likely to be bilateral than by chance, χ^2 ^= 8.3, df = 1, p < 0.01

**Table 6:** Percent of 249 indoor and 39 outdoor housed lactating sows from 86 English farms with new body lesions score 0-3 by location

	Shoulders		Hips		Tail		Spine	
Score^1^	Indoor	Outdoor	Indoor	Outdoor	Indoor	Outdoor	Indoor	Outdoor
Score 0	78.7	94.9	96.0	94.9	87.6	97.4	90.0	100.0
Score 1	13.3	2.6	2.4	2.6	9.2	0.0	7.6	0.0
Score 2	7.2	2.6	1.2	0.0	2.4	2.6	2.0	0.0
Score 3	0.8	0.0	0.4	2.6	0.8	0.0	0.4	0.0

Total n	249	39	249	39	249	39	249	39

^1^0 = no lesion, 1 = redness/soreness where the surface of the skin is not broken or a small area of broken skin/scab (<2 cm), 2 = moderate area of broken skin/scab (2-3 cm), 3 = large area of broken skin/scab (>3 cm)

**Table 7:** Number and percent of lactating sows from 86 indoor and outdoor farms in England with limb lesions by floor type, week of lactation, responsiveness, space in the crate, slat material and bedding type

	Wounds		Calluses		Bursitis		Capped hock		Total n
	n	%	n	%	n	%	n	%	
Floor/bedding									
Outdoor	0	0.0	13	37.1	4	11.4	11	31.4	35
Solid with bedding	5	14.7	30	88.2	13	38.2	17	50.0	34
Partly slatted with bedding	4	7.8	47	92.2	24	47.1	30	58.8	51
Partly slatted no bedding	32	25.8	112	90.3	50	40.3	80	64.5	124
Fully slatted	10	28.6	35	100.0	12	34.3	21	60.0	35
Week of lactation									
1-week	19	26.0	64	87.7	20	27.4	37	50.7	73
2-week	17	21.8	65	83.3	26	33.3	44	56.4	78
3-week	9	14.1	52	81.3	27	42.2	36	56.3	64
4-week	6	9.4	56	87.5	30	46.9	42	65.6	64
Sow's responsiveness to humans^1^									
Bright, alert and responsive	28	15.9	141	80.1	57	32.4	100	56.8	176
May be dull	17	21.3	76	95.0	33	41.3	45	56.3	80
Dull and unresponsive	6	26.1	20	87.0	13	56.5	14	60.9	23
Space between sow's back and crate									
<5 cm	5	16.1	30	96.8	15	48.4	24	77.4	31
5-10 cm	15	26.3	50	87.7	21	36.8	35	61.4	57
>10 cm	27	19.9	125	91.9	55	40.4	82	60.3	136
Space between sow's tail and crate									
<10 cm	7	20.0	31	88.6	18	51.4	22	62.9	35
10 - 20 cm	11	17.5	57	90.5	30	47.6	39	61.9	63
>20 cm	28	23.0	113	92.6	41	33.6	77	63.1	122
Slat material									
Metal	23	22.7	90	89.1	50	49.5	56	55.4	101
Plastic	20	20.6	92	94.8	32	33.0	65	67.0	97
Metal and plastic	3	27.3	11	100	3	27.3	8	72.7	11
Bedding type									
Straw	7	10.9	58	90.6	25	39.1	36	56.3	64
Wood shavings	8	25.8	27	87.1	16	51.6	20	64.5	31
Paper	1	16.7	6	100.0	2	33.3	3	50.0	6

^1^[12]

**Table 8:** Number and percent of lactating sows from 86 farms in England with limb lesions by floor type, week of lactation, responsiveness, space in the crate and slat material

	Shoulders		Hips, spine, tail		Total n
	n	%	n	%	
Floor/bedding					
Outdoor	1	2.6	0	0.0	39
Solid with bedding	2	5.9	5	14.7	34
Partly slatted with bedding	8	15.7	4	7.8	51
Partly slatted no bedding	13	10.5	16	12.9	124
Fully slatted	5	15.6	7	21.9	32
Week of lactation					
1-week	15	23.8	11	17.5	63
2-week	5	7.4	8	11.8	68
3-week	4	7.1	3	5.4	56
4-week	4	7.4	10	18.5	54
Sow's responsiveness to humans^1^					
Bright, alert and responsive	9	6.2	21	14.5	145
May be dull	12	15.4	9	11.5	78
Dull and unresponsive	7	38.9	2	11.1	18
Space between sow's back and crate					
<5 cm	7	22.6	7	22.6	31
5-10 cm	11	19.6	8	14.3	56
>10 cm	9	6.7	13	9.6	135
Space between sow's tail and crate					
<10 cm	8	24.2	9	27.3	33
10 - 20 cm	7	11.5	6	9.8	61
>20 cm	12	9.7	13	10.5	124
Slat material					
Metal	14	11.2	19	15.2	125
Plastic	16	14.7	14	12.8	109
Metal and plastic	2	18.2	0	0.0	11
Bedding type					
Straw	7	10.9	9	14.1	64
Wood shavings	4	13.3	1	3.3	30
Paper	2	33.3	0	0.0	6

^1^[12]

**Table 9:** Two level binomial logistic regression models of the risks associated with limb lesions in lactating sows from 82 indoor farms in Britain

	Wounds n = 267		Calluses n = 255		Bursitis n = 276		Cap. hock n = 268	
Intercept coefficient	0.3		2.2		1.1		-4.0	
	OR	CI	OR	CI	OR	CI	OR	CI
Week of lactation	0.6	0.4, 0.8*	1.3	1.0, 1.8	1.5	1.2, 1.8*	1.2	0.9, 1.5
Body condition score	0.7	0.4, 1.4	0.6	0.3, 1.2	0.4	0.2, 0.7*	1.8	1.0, 3.4
Floor/bedding								
Solid with bedding								
Partly slatted with bedding	1.0	0.2, 4.5	3.9	1.1, 13.4*	1.8	0.7, 4.8	1.4	0.4, 4.9
Partly slatted no bedding	3.2	0.9, 12.1	4.4	1.4, 13.4*	1.2	0.5, 2.8	3.5	1.2, 10.7*
Fully slatted	5.7	1.2, 26.6*	8.9	2.0, 38.6*	0.8	0.3, 2.3	3.8	1.04, 14.2*
Initial response to human presence^1^								
Bright alert and responsive								
May be dull					1.2	0.7, 2.1	2.3	1.2, 4.0*
Dull and unresponsive					2.7	1.1, 7.0*	2.3	0.9, 6.3
Space between sow's back and the crate								
<5 cm								
5-10 cm			0.2	0.1, 0.9*				
>10 cm			0.2	0.1, 0.8*				
Dry slurry in the sow lying area								
No								
Yes	0.2	0.05, 0.8*					0.2	0.1, 0.6*
Random effects	Var.	SE	Var.	SE	Var.	SE	Var.	SE
Variation between farms	0.9	0.4	0.7	0.4	1.1	1.0	0.4	0.3
Hosmer-Lemeshow goodness-of-fit	χ^2^	P value	χ^2^	P value	χ^2^	P value	χ^2^	P value
	8.1	0.15	1.3	0.93	3.3	0.66	3.8	0.60

^1^[12]

*CI does not include unity therefore the factor is significantly different from the reference category (p < 0.05)

**Table 10:** Two level logistic regression models of the risks associated with body lesions in 249 lactating sows from 80 indoor farms in Britain

	Shoulders		Hips, spine or tail	
Intercept coefficient	0.9		1.2	
	OR	CI	OR	CI
Week of lactation	0.7	0.4, 1.0	1.0	0.7, 1.4
Body condition score	0.5	0.2, 1.3	0.5	0.2, 1.2
Floor/bedding				
Solid with bedding				
Partly slatted with bedding	0.9	0.1, 6.1	0.5	0.1, 3.2
Partly slatted no bedding	1.0	0.2, 5.6	1.3	0.3, 5.9
Fully slatted	1.7	0.2, 12.3	4.7	0.9, 25.0
Initial response to human presence^1^				
Bright alert and responsive				
May be dull	3.4	1.3, 8.6*	1.0	0.4, 2.3
Dull and unresponsive	4.8	1.2, 19.6*	0.7	0.1, 4.0
Space between sow's tail and the crate				
<10 cm				
10-20 cm	0.5	0.1, 1.7	0.2	0.1, 0.6*
>20 cm	0.3	0.1, 0.9*	0.2	0.1, 0.7*
Wet slurry in the sow lying area				
No				
Yes	7.1	1.5, 34.1*		
Cracked/broken floor in the sow lying area				
No				
Yes	4.7	1.1, 19.5*		
Random effects	Var.	SE	Var.	SE
Variation between farms	0.5	0.6	0.6	0.6
Hosmer-Lemeshow goodness-of-fit	χ^2^	P value	χ^2^	P value
	1.7	0.92	6.4	0.27

^1^[12]

*The CI does not include unity therefore the factor is significantly different from the reference category (p < 0.05)

**Table 11:** Pearson correlation coefficients for limb and body lesions in 339 indoor and outdoor lactating sows from 101 farms in Britain

		Shoulder lesions		Hip, spine or tail lesions		Bursitis	Calluses	Wounds on limbs
		New	Scars	New	Scars			
Shoulder lesions	New	1.00						
	Scars	-0.03	1.00					
Hip spine tail lesions	New	0.05	-0.03	1.00				
	Scars	-0.03	-0.02	-0.02	1.00			
Bursitis		0.01	-0.01	0.16**	0.08	1.00		
Calluses		0.18**	0.09	0.12*	-0.06	0.12*	1.00	
Wounds on limbs		0.08	0.06	0.09	-0.03	0.14*	0.17**	1.00
Capped hock		0.09	0.04	0.02	0.09	0.02	0.18**	0.02

* significantly correlated at p < 0.05, ** significantly correlated at p < 0.01

**Table 12:** Population attributable fractions of limb and body lesions in indoor and outdoor housed lactating sows from 86 farms in England

	Limb lesions n = 279				Body lesions n = 288	
Flooring types	Wound	Callus	Bursa	Capped hock	Shoulder	Hip, spine and tail
Outdoor						
Solid with bedding	9.8	7.3	8.8	4.0	5.8	5.5
Partly slatted with bedding	7.8	11.8	17.6	8.8	14.8	14.9
Partly slatted no bedding	62.7	9.3	34.8	25.8	37.1	29.2
Fully slatted	19.6	9.3	7.8	6.3	14.2	11.7
Total reduction	100.0	56.3	69.0	44.9	71.9	61.3

All sows kept outdoors were housed in huts set on soil with deep straw bedding and access to an outdoor paddock. Of the 278 sows housed indoors on 82 farms, 12.2% (34) were kept on solid concrete floors with bedding, 21.6% (60) on partly slatted floors with bedding, 51.8% (144) on partly slatted floors without bedding and 14.4% (40) on fully slatted floors. During the previous gestation all outdoor housed sows were housed outdoors on soil, 10% of the indoor housed sows were housed on slatted floors and 90% were housed on solid concrete floors with bedding. Body condition score ranged from 1.5 to 4 with a mean of 2.9 (SD 0.5), 88% of the sows were score 2.5-3.5. The sows' parity ranged from 1-14 with a median of 3 (IQR 2, 4). Parity did not differ significantly between indoor and outdoor housed sows (U = 0.37, p = 0.71). However, parity was unknown for 20.0% of the sows.

### Prevalence of limb and body lesions

The prevalence of wounds, calluses, bursitis and capped hock on the limbs of 279 sows was 18.3% (51), 84.9% (237), 36.9% (103) and 57.0% (159) respectively. Wounds and bursitis were more prevalent on the hind limbs whilst calluses were more prevalent on the fore limbs. The prevalence of lesions on the right and left limbs was very similar and all limb lesions were significantly more likely to be bilateral then by chance (Table 4). The modal maximum lesion score was one for wounds and capped hock and two for calluses and bursitis (Table 4). The farm level prevalence of wounds, calluses, bursitis and capped hock was 37.6%, 94.1%, 71.8% and 85.9% respectively.

The prevalence of scars and new body lesions in 288 indoor and outdoor housed sows was 35.4%; 17.7% of sows had scars and 19.8% had new lesions. The prevalence of new lesions on the shoulders was 10.4% and the prevalence elsewhere on the body (hip, spine or tail area) was 11.7% (Table 5). The farm level prevalence of body lesions overall was 66.7%.

Body lesions at all locations were more prevalent and larger (higher score) in sows housed indoors compared with sows housed outdoors (Table 6).

Only one of the 39 sows housed outdoors had a new body lesion, the rest had old scars. The prevalence of limb and body lesions varied by floor type, floor condition, space in the crate, sow body condition and responsiveness to humans (Tables 7 and 8).

### Risk factors associated with limb lesions in lactating sows

#### Wounds

No wounds on the limbs were observed in outdoor housed lactating sows. In indoor housed sows the risk of wounds on the limbs reduced with increasing week of lactation. There was an increased risk of wounds on the limbs associated with fully slatted floors, and a non significant trend for an increased risk on partly slatted floors with no bedding, compared with solid concrete floors with bedding. There was a reduced risk of wounds on the limbs on floors with dry slurry in the sow lying area (Table 9).

#### Calluses

There was a reduced risk of calluses in lactating sows housed outdoors compared with sows housed indoors (OR 0.1, CI 0.1, 0.2). In lactating sows housed indoors, there was a trend for the risk of calluses to increase with increasing week of lactation but the CI included unity. There was an increased risk of calluses in sows housed indoors on partly slatted floors with bedding, partly slatted floors with no bedding and fully slatted floors compared with solid concrete floors with bedding. There was a decreased risk of calluses in sows in crates with 5 - 10 cm, or more than 10 cm between the sows back and the top of the crate compared with sows with less than 5 cm (Table 9).

#### Bursitis

There was a reduced risk of bursitis associated with sows housed outdoors compared with sows housed indoors (OR 0.2, CI 0.1, 0.6). In sows housed indoors, the risk of bursitis increased with increasing week of lactation and decreased with increasing sow body condition. Sows that were 'dull and unresponsive' to human presence had an increased risk of bursitis compared with sows that were 'bright, alert and responsive' (Table 9).

#### Capped hock

There was a reduced risk of capped hock associated with sows housed outdoors compared with sows housed indoors (OR 0.1, CI 0.1, 0.6). Among indoor housed sows, an increased risk of capped hock was associated with partly slatted floors with no bedding and fully slatted floors compared with solid concrete floors with bedding. Sows that were classified as 'may be dull' had an increased risk of capped hock compared with sows that were 'bright, alert and responsive'. There was a trend for an increased risk of capped hock with increasing body condition, but the CI included unity. A reduced risk of capped hock was associated with floors with a covering of dry slurry in the sow lying area (Table 9).

### Risk factors associated with body lesions in lactating sows

#### Body lesion scars

There was a positive association between body lesion scars and the parity of the sow (OR 1.2, CI 1.1, 1.4). No other associations were detected between floor type, crate size or sow behaviour and body lesion scars.

#### New shoulder lesions

There was only one outdoor housed sow with a new shoulder lesion, so there were insufficient data to calculate an estimate of risk between the prevalence outdoors (2.4%) and indoors (12.1%). Indoors, there was a trend for a decreasing risk of new shoulder lesions with increasing week of lactation, but the CI included unity. The risk of new shoulder lesions reduced as the space between the sow's tail and the back of the crate increased and there was a non significant trend for a decreased risk of new shoulder lesions with increasing body condition. The risk of new shoulder lesions increased as responsiveness to human presence decreased. An increased risk of new shoulder lesions was also associated with a lying area that was damaged or had a covering of wet slurry compared with clean, dry, undamaged floors (Table 10).

#### New hip, spine and tail lesions

None of the outdoor housed sows in the risk factor analysis had a new lesion on the hip, spine or tail. In indoor housed lactating sows the risk of new lesions on the hips, spine or tail reduced as the space within the crate increased. There were non significant trends for an increased risk of hip, spine and tail lesions associated with fully slatted floors, compared with solid concrete floors, and a decreased risk with increasing body condition (Table 10).

#### Model fit and observer differences

**Figure 1:**
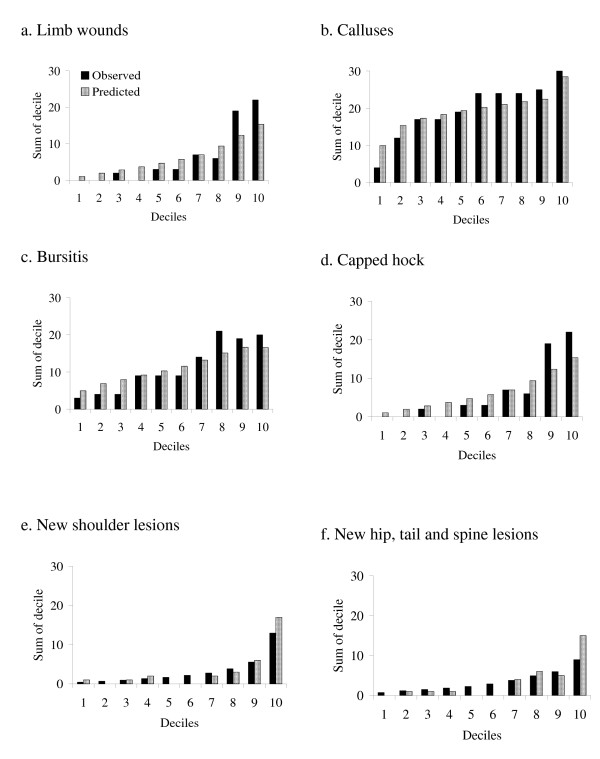
**Observed verses predicted values for limb and body lesion models in lactating sows**.

The Hosmer-Lemeshow goodness-of-fit statistic indicated that the differences between the observed and predicted values in the models were generally small (Tables 9 and 10). Graphs of predicted verses observed values illustrated that there was a trend for the models to under predict the prevalence of limb lesions but over predict the prevalence of body lesions in the higher deciles (Figure 1). Controlling for observer or time of year the data were collected did not alter the interpretation of the fixed effects in any models.

#### Associations between limb and body lesions and slat materials and bedding type

Having accounted for floor type there were no significant associations between slat material (metal or plastic) or bedding type (wood shavings or straw) and the prevalence of any limb or body lesions in indoor housed lactating sows (data not shown).

#### Correlations between lesions

At a significance level of p < 0.01, calluses were positively correlated with wounds on the limbs, capped hock and new shoulder lesions. New lesions on the hip, spine or tail were positively correlated with bursitis. Additional correlations at a 5% significance level are presented in Table 11. Overall the R values were low and explained less than 20% of the variation between the paired variables (Table 11).

#### Correlations between parity, body condition and crate space

As the parity of the lactating sow increased, the body condition increased (r = 0.20, df = 219, p < 0.05) and the space in the crate decreased (between the sow's back and the top of the crate; r = -0.37, df = 219, p < 0.05, between the sow's tail and the back of the crate; r = -0.28, df = 219, p < 0.05). Sows that had less space within the crate between the sow's tail and the back of the crate were less likely to respond to human presence (r = 0.13, df = 278, p < 0.05).

#### Population attributable fractions

Based on the association between floor type and limb and body lesions reported in the current study, the prevalence of injuries in the diseased population would be reduced by between 32% and 100% (depending on the type of lesion, see 'total reduction' in Table 12) if sows currently housed indoors during lactation were housed outdoors. This also assumes that sows would be housed outdoors during pregnancy as for the outdoor housed lactating sows in the current study. For all types of limb and body lesions the largest proportion of lesions was attributable to part slatted floors with or without bedding (Table 12).

## Discussion

This current study is, to the authors' knowledge, the first cross-sectional study of the prevalence of limb and body lesions in lactating sows on commercial English pig farms. Previous studies have been conducted in other countries and carried out on experimental units or with small numbers of farms or sampled a different population, e.g. culled sows or included pregnant sows [1-3,6,9]. As such the current study provides a useful benchmark for future work.

The farms in the current study are thought to be a reasonable representation of the English pig farm population in 2003-2004. The spatial location of farms reflects the location of pig farms in England with more farms sampled from the pig dense areas of North Yorkshire and East Anglia [17]. While only 17% of the study farms housed pigs outdoors, these farms housed approximately 30% of the breeding sows in the study herds, which was similar to the indoor: outdoor pig ratio in the British herd in June 2003 [18]. The mean herd size of the study farms (409 sows) was larger than the mean herd size (220 sows) of farms fitting the selection criteria in the national herd in 2003 (DEFRA June 2003 statistics, personal communication) but this might reflect the fact that herd sizes were increasing in Britain during this period [18]. It is possible that larger herds might have biased the data towards newer floors and more intensive systems. This could affect the prevalence estimates but would not have an impact on the associations between floors and lesions presented.

Assured British Pigs was the best sampling frame available, reportedly representing 90% of the national herd [19]. Sampling members of a farm assurance scheme might have resulted in a bias for farms with higher health and welfare standards. However, due to the high coverage of ABP, much of the variation in the population is captured within the sampling frame. Compliance in the current study was voluntary, which again may have biased the sample towards motivated farmers with higher standards. This could mean that the prevalence values presented from the current study underestimate the prevalence of limb and body lesions in the national herd but it is unlikely that this would bias associations between exposures and outcomes.

The number of sows in the prevalence calculations, 279 for limb lesions and 288 for body lesions, was less than the 400 specified in the study design as we did not manage to recruit 100 farms, and did not collect full data from four sows on all farms. This is likely to have increased the error around the prevalence estimates from 5% to around 7%. The sample size for prevalence calculations of lactating sows housed outdoors was low (n = 35-41 depending on outcome, from 16 farms). However, this study does provide useful information on which further work might be based. The sample size used in the risk factor analysis was sufficient to detect a 3 fold difference in risk between the exposed and unexposed sows and many useful hypotheses were generated.

The prevalence of limb and body lesions in indoor housed lactating sows was high, while the prevalence in sows housed outdoors was significantly lower (where tested). It is likely that soil and deeply bedded lying areas protect the sows from limb and body lesions. A similar pattern was reported in these sows' preweaning piglets [20]. Although analysis of a larger sample would be required to be confident this finding applied to all sows on outdoor farms, the association between injuries and slatted and unbedded floors makes this a plausible hypothesis for future studies.

As in previous studies [1,3,4,9], there was an overall trend for the risk of calluses, capped hock and limb wounds to increase as the proportion of slatted floor in the farrowing crate increased and the quantity of bedding decreased. The risk of bursitis did not differ with floor type, however the prevalence of bursitis increased the longer the sow had been in the farrowing crate, suggesting that all floor surfaces were sufficiently hard to cause bursae to develop.

In addition to the effect of floor type, there was an increased risk of bursitis, capped hock and shoulder lesions in sows that were less responsive to human presence (classified as 'may be dull' or 'dull and unresponsive'). This might occur because these sows spent more time lying down because rising was difficult, e.g. slippery floors, or because once sows develop these lesions, they may experience sufficient discomfort to discourage them from rising to their feet.

It is unclear why a film of dry slurry on the floor reduced the risk of wounds on the limbs and capped hock. It is possible that the coating of dry dung made the floor less slippery, less abrasive or less hard or that a coating of dung on the sows' limbs made it difficult to detect lesions. It is possible that there might be misclassification of sows housed outdoors for this same reason. To eliminate this source of error it would be necessary to clean each sow before examination. In this cross sectional study time constraints meant that this was not feasible.

Sows that had less space in the crate had an increased risk of body lesions and calluses on the limbs, possibly because they were more likely to injure themselves against the crate whilst standing [5] or because it is harder for them to change position and move from lying to standing. Higher parity was correlated with higher body condition and therefore older sows had less space in the crate and were at increased risk of developing body lesions and calluses. Parity was not included in the final models because of the co-linearity between these variables and because parity is not a useful variable when considering intervention strategies (parity will increase in all breeding sows). The latter two explanatory variables suggest that larger sows should be kept in larger crates to minimise risk of injury. There were probably no detectable correlations between scars of old body lesions and the sow's current environment because some of these scars were from lesions that occurred in previous lactations.

There might be a reduced risk of bursitis with increasing body condition because subcutaneous fat protects against bursitis [21] or makes bursitis harder to detect. Conversely, there was a trend for increased body condition to be associated with an increased risk of capped hock. This might have occurred because heavier or older sows are at increased risk of capped hock but it can not be ruled out that there might have been a misclassification of sows with fatty hocks. The different risks for the two lesions suggests that, as proposed for finishing pigs [14], capped hock and bursae are aetiologically distinct.

As reported in several previous studies [4,6-9], there was a trend for the risk of wounds on the limbs and body to increase in sows with poorer body condition, where there is less subcutaneous fat over the bony protuberances. This might not have reached significance in the current study because the majority of sows in this sample were in good body condition therefore a larger sample would be required to detect a significant effect. We propose that the good body condition of lactating sows arises from the good body condition of loose group-housed gestating sows that have to be fed well to avoid bullying. Sow body condition in the British breeding herd appears to be higher and more uniform than 10 - 15 years ago (Green, personal observation). Wet and rough floors probably increased the risk of shoulder sores, as reported in previous studies [4,6,9,22], because moisture softened the skin and the rough floor surface was abrasive.

The prevalence of wounds on the limbs and body was highest in the first week of lactation in indoor housed sows (no sows were examined until three days post partum by which time they are likely to have been in the crate for around eight days). This might have occurred because when sows first entered the crate they were unaccustomed to having their movement restricted and this increased the risk of injury [23,24] and also sows spent a large amount of time lying recumbent during the first days post partum [9,22].

## Conclusion

The sample of outdoor housed sows in this study had the lowest prevalence of limb and body lesions. In sows housed indoors there was a general trend for an increased risk of limb and body lesions in lactating sows housed on slatted floors compared with those housed on solid concrete floors with bedding. Sows that were less responsive to human presence and sows that had the least space to move within their crates had an additional increased risk of developing these lesions.

## Abbreviations

The following abbreviations were used in this paper; (OR): Odds ratio; (CI): Confidence interval; (IQR): Interquartile range; (SE): Standard error; (Var.): Variance; (ABP): Assured British Pigs; (DEFRA): Department for Environment, Food and Rural Affairs

## Competing interests

The authors declare that they have no competing interests.

## Authors' contributions

ALK: participated in the study design, data collection and data management, carried out the data analysis and drafted the manuscript. CEG: provided advice and assistance with data analysis. LEG: conceived the project, designed the study, oversaw project management and supervised statistical analysis and assisted with preparation of the manuscript. All authors have read and contributed to the final draft of the manuscript.
